# Sex-dependent regulation of vertebrate somatic growth and aging by germ cells

**DOI:** 10.1126/sciadv.adi1621

**Published:** 2024-06-12

**Authors:** Kota Abe, Hikaru Ino, Tomomi Niwa, Daniel Semmy, Ayami Takaochi, Takashi Nishimura, Chihiro Mogi, Maki Uenaka, Masaru Ishii, Kaori Tanaka, Yasuyuki Ohkawa, Tohru Ishitani

**Affiliations:** ^1^ Department of Homeostatic Regulation, Division of Cellular and Molecular Biology, Research Institute for Microbial Diseases, Osaka University, Suita, Japan.; ^2^ Metabolic Regulation and Genetics, Department of Molecular and Cellular Biology, Institute for Molecular and Cellular Regulation, Gunma University, Maebashi, Japan.; ^3^ Institute for Molecular and Cellular Regulation, Gunma University, Maebashi, Japan.; ^4^ Department of Immunology and Cell Biology, Graduate School of Medicine / Graduate School of Frontier Biosciences, Osaka University, Suita, Japan.; ^5^ Immunology Frontier Research Center, Osaka University, Suita, Japan.; ^6^ Center for Infectious Disease Education and Research (CiDER), Osaka University, Suita, Japan.; ^7^ Division of Transcriptomics, Medical Institute of Bioregulation, Kyushu University, Fukuoka, Japan.

## Abstract

The function of germ cells in somatic growth and aging has been demonstrated in invertebrate models but remains unclear in vertebrates. We demonstrated sex-dependent somatic regulation by germ cells in the short-lived vertebrate model *Nothobranchius furzeri*. In females, germ cell removal shortened life span, decreased estrogen, and increased insulin-like growth factor 1 (IGF-1) signaling. In contrast, germ cell removal in males improved their health with increased vitamin D signaling. Body size increased in both sexes but was caused by different signaling pathways, i.e., IGF-1 and vitamin D in females and males, respectively. Thus, vertebrate germ cells regulate somatic growth and aging through different pathways of the endocrine system, depending on the sex, which may underlie the sexual difference in reproductive strategies.

## Teaser

Germ cell removal affects the life span and body size of vertebrates in a sex-dependent manner.

## INTRODUCTION

Life history theory, a principle of evolutionary biology, suggests that reproduction increases the costs of somatic investment (*1*–*3*). Similarly, a possible trade-off between germ cells and the growth and maintenance of the soma, the disposable soma theory, is a central tenet of the biology of aging (*4*–*6*). Although studies to date strongly support the existence of this trade-off (*7*, *8*), it is unclear whether the trade-off is the direct result of conflicts in resource allocation or stems from molecular signals that inversely regulate somatic and reproductive investments (*2*). The existence of the latter trade-off has been suggested in studies on *Caenorhabditis elegans*. Ablation of germline precursor cells extends the life span of *C. elegans*, but ablation of the entire gonad has no effect, suggesting that gonad-dependent signals influence life span (*9*, *10*). The specific ablation of germ cells also extends the life span of *Drosophila melanogaster* (*11*). However, the effect of germ cell removal on vertebrate somas remains largely unclear.

Given that somatic growth and aging are inevitably linked, the interaction between reproduction and the soma becomes more complex. In *C. elegans*, germline ablation increases both growth and life span (*2*). However, from a life history perspective, the more organisms invest in growth, the less they invest in soma maintenance (*12*, *13*). An inverse correlation between growth and life span has been reported in many vertebrate species, including fish, reptiles, and mammals (*14*–*16*). Accordingly, the interaction between the three factors, reproduction, somatic growth, and aging, and the molecular basis for this connection remain largely unexplored. In particular, the effects of germ cell removal on somatic growth and aging in vertebrates are poorly understood, hampered by the long life span of available model organisms such as mice, zebrafish, and medaka, although one recent study in zebrafish suggested that germ cell removal improves somatic regeneration (*6*). To address this question, we conducted germ cell removal in a short-lived (several months of life span) and fast-growing (sexual maturation in approximately one month) vertebrate model, the African turquoise killifish (*Nothobranchius furzeri*) (*17*, *18*).

## RESULTS

### Germ cell removal affects the life span of *N. furzeri* in a sex-dependent manner

**Fig. 1.**
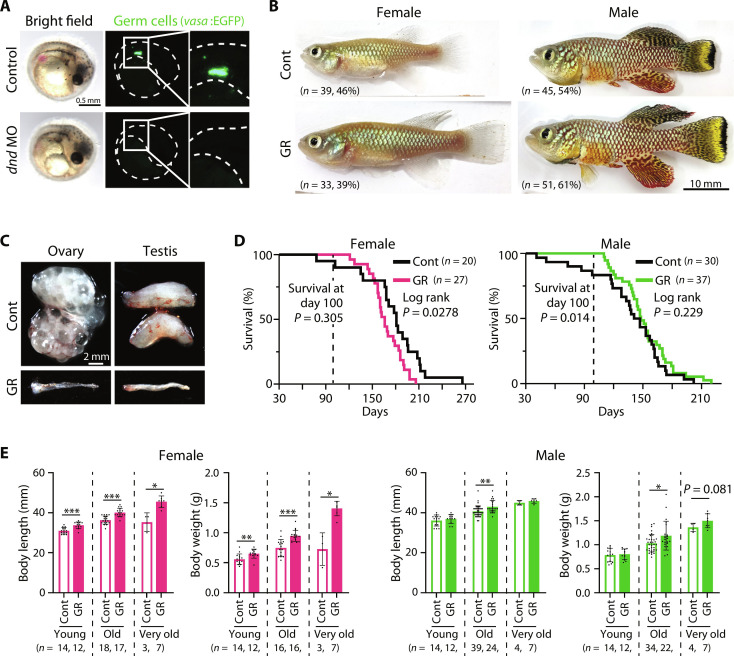
Germ cell removal modulates life span and body size in *N. furzeri*. (**A**) Germ cell–removed *N. furzeri* embryo by injecting morpholino antisense oligo (MO) against *dnd* at 12 days postfertilization. Germ cells were visualized by EGFP expression in Tg (*vasa:EGFP*) transgenic strain. (**B**) Adult control (Cont) and germ cell–removed *N. furzeri* (GR)*.* (**C**) Gonads of control (Cont) and germ cell–removed *N. furzeri* (GR)*.* (**D**) Life span of control and germ cell–removed *N. furzeri*. Left: Survival curves of control (Cont, black) versus germ cell–removed female (GR, magenta). Right: Survival curves of control (Cont, black) versus germ cell–removed male (GR, green). (**E**) Quantifications of body length and body weight of control (Cont) and germ cell–removed (GR) *N. furzeri*. Animals measured at each time point are different individuals. *P* values are from Welch’s *t* test (*P < 0.05; **P < 0.01; ***P < 0.001). Bars and error bars represent mean ± SD.

To investigate the role of germ cells in the regulation of somatic growth and aging in *N. furzeri*, we injected morpholino antisense oligonucleotides (MOs) to knock down the germline-specific gene *dead end* (*dnd*) (fig. S1, A and B). The *dnd* gene is essential for the normal migration and survival of primordial germ cells; therefore, knockdown of *dnd* resulted in the production of germ cell–removed animals without observable side effects on somatic tissues (*6*, *19*). To check whether germ cells were removed, we generated a Tg (*vasa:EGFP*) transgenic line with an enhanced green fluorescent protein (EGFP) driven by the germ cell–specific promoter, *vasa* (*20*). Using *vasa*:*EGFP*, we confirmed that germ cells were lost in *dnd* knockdown morphants at 12 days postfertilization when organogenesis was nearly complete (Fig. 1A) (*21*). These germ cell–depleted embryos developed into fish that appeared phenotypically as males and females, as determined by their body color (Fig. 1B). When males mated with control females, they displayed normal mating behavior. Germ cell–depleted fish of both sexual phenotypes had gonads with tube-like structures (Fig. 1C).

Using this method, we examined the effect of germ cell removal on life span. In females, germ cell removal significantly reduced life span (10% decrease in median survival and 6.6% decrease in mean survival) (Fig. 1D). In contrast, in the germ cell–depleted males, the survival rate at old age (100 days posthatching) was significantly increased, the median survival was increased by 3.5%, and mean survival was increased by 13% (Fig. 1D). These effects of germ cell removal on life span were confirmed by another *dnd* MO (figs. S1, A, C, and D, and S2A). These results indicate that vertebrate germ cells play a role in the regulation of life span; however, unlike invertebrate models (*9*, *11*), their roles differ in a sex-dependent manner.

### Germ cell removal increases the body growth of *N. furzeri*

We also examined the effects of germ cell removal on body growth at young (one and a half months: age at sexual maturation), old (three months: age approximately at the 75% survival in wild type), and very old (four and a half months: age approximately at the 25% survival in wild type) ages. In females, germ cell removal significantly increased body length and weight by 10 and 27%, respectively, in old age (Fig. 1, B and E). Although control females hardly grew after old age, germ cell–depleted females continued to grow until they were at very old age (Fig. 1E). In males, body length and weight were increased by 5.7 and 16%, respectively, at old age (Fig. 1, B and E). Both control and germ cell–removed males continued growing until very old age (Fig. 1E). The increase in the body size was confirmed by another *dnd* MO (fig. S2B). These results suggest that germ cells negatively regulate the somatic growth rates in both males and females.

### Germ cell removal does not cause sex reversal but affects steroidogenesis in *N. furzeri*

**Fig. 2.**
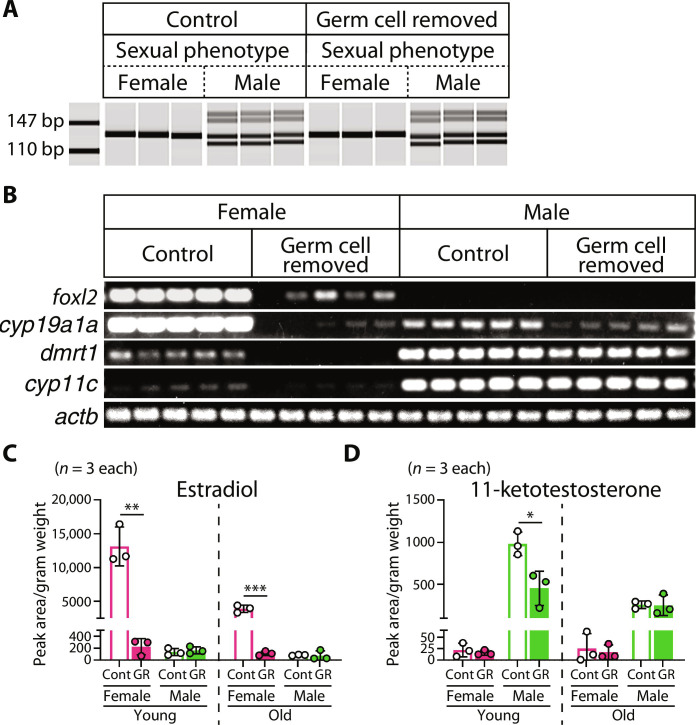
Germ cell removal does not cause sex reversal but affects steroidogenesis in *N. furzeri.* (**A**) Heteroduplex mobility assay for genotyping of *gdf6*, the sex-determining gene of *N. furzeri*. In genotypic female, a single 134-bp PCR product from X chromosome is detected. In genotypic male, 125-bp PCR product from Y chromosome is mixed, and thus four bands including two kinds of hetero dimers are detected. (**B**) RT-PCR analysis of male markers, *dmrt1* and *cyp11c*, and female markers, *foxl2* and *cyp19a1a* in adult gonads at young age, *actb* is used as internal control. (**C** and **D**) Quantification of (C) estradiol and (D) 11-ketotestosterone levels extracted from the whole body of *N. furzeri* at young and old age. Cont, control. GR, germ cell removed. *P* values are from Welch’s *t* test (*P < 0.05; **P < 0.01; ***P < 0.001). Bars and error bars represent mean ± SD.

To examine how these life course events are affected by germ cell removal, we focused on sexual differentiation. Sex is an important factor associated with life span and growth (*22*). Germ cell–depleted embryos develop into adult fish with external male phenotypes in several teleost species, including zebrafish and medaka (*19*, *23*). Therefore, we first examined whether external sexual characteristics coincided with genotypic sex. The sex-determining gene of *N. furzeri* is *gdf6*, and *gdf6* on the Y chromosome differs from *gdf6* on the X chromosome by 22 single-nucleotide variants and a 9-bp deletion (*17*). By genotyping to detect the 9-bp deletion, we found that external sexual characteristics, judged by body color, did not differ from genotypic sex in *N. furzeri* (Figs. 1B and 2A). Next, we examined the sexual characteristics of the gonads. Reverse transcription polymerase chain reaction (RT-PCR) revealed that germ cell removal did not increase the expression of male genes (*dmrt1* and *cyp11c*) in female gonads (Fig. 2B). Similarly, it did not up-regulate female gene expression (*foxl2* and *cyp19a1a*) in male gonads (Fig. 2B). These results indicated that germ cell removal does not affect sex determination in *N. furzeri*. Notably, the expression of female genes, especially *cyp19a1a*, which encodes estrogen synthetase, was substantially reduced in the gonads of germ cell–removed females (Fig. 2B), suggesting that steroid hormone production was abrogated. Consistently, mass spectrometry analysis revealed that estradiol levels were drastically reduced in germ cell–removed females (Fig. 2C). We also confirmed that germ cell removal did not reverse the levels of sex steroids but reduced the level of 11-ketotestosterone in males at a young age (Fig. 2, C and D). These data suggest that the germ cells of *N. furzeri* are not determinants of sexual fate but affect the production of sex hormones, especially in females.

### Germ cell–removed females exhibit altered health status associated with reduced estrogen signaling

**Fig. 3.**
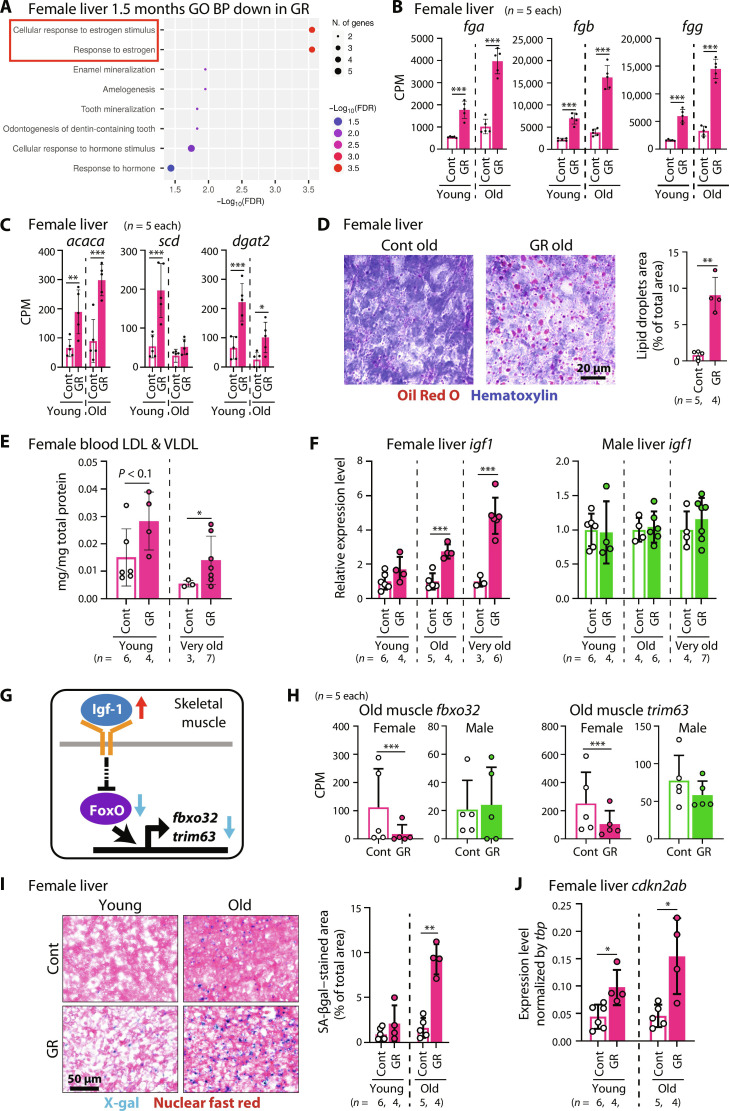
Low estrogen and increased IGF-1 signaling is associated with accelerated aging in germ cell–removed females. (**A**) GO term analysis of down-regulated genes in the female livers. Red box indicates “cellular response to estrogen stimulus” and “response to estrogen.” N. of genes, number of genes. BP, biological process. (**B**) Expression levels of fibrinogens; *fga*, *fgb*, and *fgg* in the female livers by RNA-seq. (**C**) Expression levels of key genes of fatty acid biosynthesis (*acaca*) and triglyceride biosynthesis (*scd* and *dgat2*) in the female liver by RNA-seq. (**D**) Liver sections of old females stained with Oil Red O and counterstained with hematoxylin. Right: Quantification of lipid accumulation in the liver. (**E**) Sum of blood LDL and VLDL levels in females. (**F**) Expression levels of *igf1* in the liver by reverse transcription–quantitative polymerase chain reaction (RT-qPCR). (**G**) Schematic illustration of IGF-1 signaling and its downstream genes in the skeletal muscle. (**H**) Expression levels of IGF-1 signaling target genes (*fbxo32* and *trim63*) in the skeletal muscle by RNA-seq. (**I**) Liver sections of females stained with senescence-associated β-galactosidase (SA-βgal) and counterstained with nuclear fast red. Right: Quantification of SA-βgal–stained area in the liver. (**J**) Expression level of cellular senescence marker *cdkn2ab* in the female livers. Cont, control. GR, germ cell removed. *P* values are from Welch’s *t* test (*P < 0.05; **P < 0.01; ***P < 0.001) in (D), (E), (F), (I), and (J). FDRs are from quasi-likelihood methods in edgeR (*FDR < 0.05; **FDR < 0.01; ***FDR < 0.001) in (B), (C), and (H). Bars and error bars represent mean ± SD. FDR, false discovery rate. CPM, count per million.

Our results suggest that germ cell removal has systemic effects on the body. To investigate these effects at the molecular level, we performed RNA sequencing (RNA-seq) on the liver and skeletal muscles, which are major organs in the body. First, we focused on the female livers. Gene Ontology (GO) term enrichment analysis of differentially expressed genes (DEGs) revealed that estrogen signaling was down-regulated by germ cell removal, specifically in females, consistent with the decreased levels of estradiol (Fig. 3A and fig. S3). In addition, coagulation-related genes, including *fga*, *fgb*, and *fgg*, were up-regulated only in females (Fig. 3B and figs. S3 and S4A). Moreover, although GO term was not significantly enriched, key genes involved in fatty acid biosynthesis and triglyceride biosynthesis (*acaca*, *scd*, and *dgat2*) were also up-regulated, specifically in females (Fig. 3C and fig. S4B). Both increased coagulation factors and abrogated lipid metabolism are observed during human menopause, a phenomenon associated with decreased estrogen levels (*24*). The increased levels of coagulation factors are believed to explain the increased risk of cardiovascular disease during menopause (*25*). Dysregulated lipid metabolism affects various aspects of energy metabolism, including adiposity and obesity (*26*). Consistent with the RNA-seq results, an increased accumulation of neutral lipids was observed in the livers of germ cell–removed females (Fig. 3D and fig. S5A). In addition, the sum of blood low-density lipoprotein (LDL) and very-low-density lipoprotein (VLDL) levels increased only in females, suggesting negative effects on the cardiovascular system (Fig. 3E and figs. S4C and S5C). These results suggest that the shortened life span of germ cell–removed females can be partly explained by the decreased estrogen levels.

### Insulin-like growth factor 1 signaling is up-regulated in germ cell–removed females

To further elucidate the mechanisms responsible for the systemic changes caused by germ cell removal, we focused on increased growth rate. Thus, we examined its effects on the insulin-like growth factor 1 (IGF-1) signaling pathway, a major regulatory pathway of body growth. Since Igf-1 is primarily produced by the liver, we examined the expression level of *igf1* in the liver. Notably, RT–quantitative PCR (qPCR) revealed that the expression of *igf1* was significantly increased in the livers of germ cell–removed females compared to that in control females, and the difference increased with age (Fig. 3F). In contrast, no significant change was observed in males despite the increased body size of germ cell–removed males (Fig. 3F), suggesting that other mechanisms may contribute to the altered growth rate in males. Next, we confirmed the increased IGF-1 signaling by focusing on skeletal muscle, the main target organ of IGF-1, based on RNA-seq. In the skeletal muscle of old females, only a small number of DEGs were detected [39 genes; false discovery rate (FDR) < 0.05]. However, representative downstream genes of IGF-1 signaling, *fbxo32* and *trim63*, whose transcription is suppressed by IGF-1 signaling in skeletal muscle (Fig. 3G) (*27*), were consistently down-regulated in germ cell–removed females (Fig. 3H). In males, the expression of these genes did not change (Fig. 3H), further confirming that the up-regulation of IGF-1 signaling by germ cell removal is specific to females.

The IGF-1 signaling is a key regulator not only of growth but also of aging. Low IGF-1 signaling activity inhibits aging partly by reducing cellular stress, including oxidative stress (*28*). This suggests that increased IGF-1 signaling may accelerate aging by promoting cellular stress. To examine the cellular stress level in tissues, we performed staining for senescence-associated β-galactosidase (SA-βgal) activity using the liver. We found that the accumulation of SA-βgal–stained cells in the liver was substantially increased in germ cell–removed females at old age compared to that in the control (Fig. 3I and fig. S5B). High activity of SA-βgal is associated with cellular senescence, the accumulation of which is an aging hallmark (*29*, *30*). Consistently, the expression level of *cdkn2ab*, which is a homolog of *p16^ink4a^*, a biomarker of cellular senescence, increased in the livers of germ cell–removed females, suggesting an accelerated accumulation of senescent cells (Fig. 3J). Together, these data suggest that germ cell removal leads to increased IGF-1 signaling activity, specifically in females, underlying increased growth rate and accelerated aging. As estrogen is reported to have antioxidant properties (*22*), reduced estrogen levels could also be responsible for increased cellular stress and cellular senescence in the liver.

### Germ cell–removed males show improved health state

**Fig. 4.**
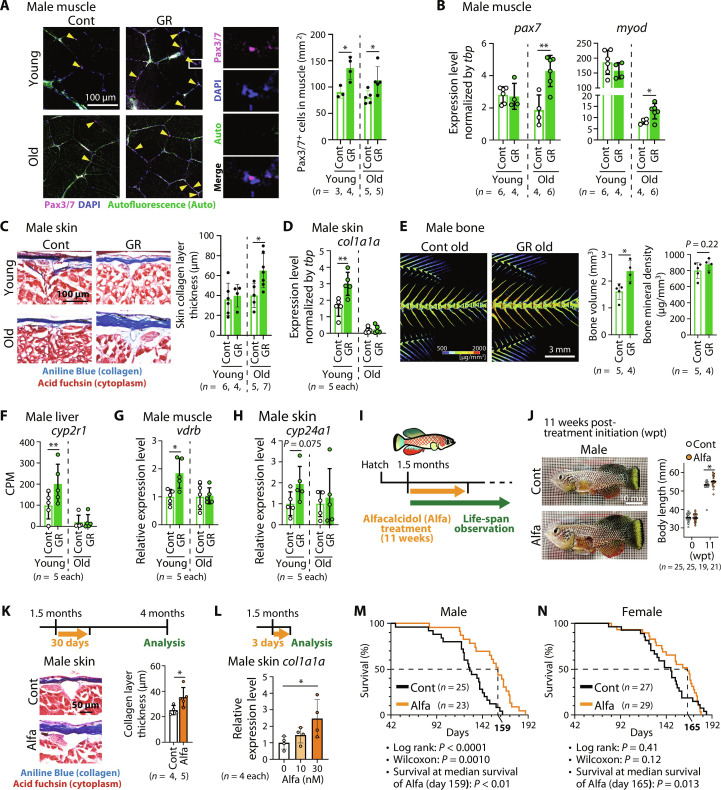
Increased vitamin D signaling contributes to somatic growth and aging prevention in germ cell–removed males. (**A**) Muscle sections of males immuno-stained with the Pax3/7 antibody (magenta), 4′,6-diamidino-2-phenylindole (DAPI) (blue), and autofluorescence (green). Yellow arrowheads indicate Pax3/7^+^ cells. Right: Quantification of Pax3/7^+^ cell number. (**B**) Expressions of satellite cell marker genes (*pax7* and *myod*) in the male skeletal muscle by RT-qPCR. (**C**) Skin sections of males stained with the Masson’s trichrome stain. Right: Quantification of the skin collagen fiber thickness. (**D**) Expression level of *col1a1a* in the male skin by RT-qPCR. (**E**) Micro-CT images of bone vertebrae of old males. Right: Quantification of the bone volume and bone mineral density. (**F**) Expression level of vitamin D activating enzyme *cyp2r1* in the male liver by RNA-seq. CPM, count per million. FDRs are from quasi-likelihood methods in edgeR (**FDR < 0.01). (**G** and **H**) Expression levels of vitamin D signaling target gene *vdrb* (skeletal muscle) (G) and *cyp24a1* (skin) (H) in males by RT-qPCR. (**I**) Schematic illustration of alfacalcidol (alfa) treatment. (**J**) Alfa-treated males at 11 weeks after treatment initiation. Right: Quantification of the body length of males at 0 and 11 weeks after treatment initiation. wpt, weeks post-treatment initiation. (**K**) Skin sections of alfa-treated males at 4 months old stained with Masson’s trichrome stain. Right: Quantification of the thickness of skin collagen fiber. (**L**) Expression of *col1a1a* in the alfa-treated male skin at a young age by RT-qPCR analysis. *P* value is determined from Dunnett’s multiple comparisons test (*P < 0.05). (**M** and **N**) Life span of control and alfa-treated males (M) and females (N). Survival curves of control (black) versus alfa-treated animals (orange). *P* values are from Welch’s *t* test (*P < 0.05; **P < 0.01) in (A), (B), (C), (D), (E), (G), (H), (J), and (K). Bars and error bars represent mean ± SD.

In contrast to females, germ cell removal appeared to improve the health state of males (Fig. 1D). To confirm this, we examined the health state of the skeletal muscle, one of the key tissues affecting the life span. It is well known that the ability of skeletal muscle to regenerate is coordinated by stem cells called satellite cells, which are compromised during aging, and the decline of satellite cells coincides with muscle loss (*31*, *32*). Notably, germ cell removal significantly increased the number of satellite cells (Fig. 4A), confirmed based on the expressions of *pax7*, a satellite cell marker, and *myod*, an activated satellite cell marker in the skeletal muscle (Fig. 4B). This could contribute to the sustained regeneration ability of the skeletal muscle.

Next, we focused on the skin, which is another important indicator of youth. During aging, dermal collagen levels decrease, which consequently leads to the deterioration of fibroblast function (*33*). Masson’s trichrome staining revealed that the thickness of the collagen fiber area increased in the skin of germ cell–removed males in old age (Fig. 4C and fig. S6). Consistently, the expression level of *col1a1a*, a major component of dermal collagen, increased in the skin of germ cell–depleted males (Fig. 4D).

In addition, we examined the bone. Micro-CT (computed tomography) analysis revealed that the bone volume was significantly increased, and bone mineral density tended to increase in germ cell–removed males (Fig. 4E). Together, these results suggest that germ cell removal improves the tissue health of males, at least in the skeletal muscle, skin, and bone.

### Vitamin D signaling contributes to somatic growth and aging prevention in the germ cell–removed male

We noticed that the expression level of *cyp2r1* increased in the livers of male germ cell–removed fish at a young age (Fig. 4F). The *cyp2r1* gene codes a major enzyme responsible for 25-hydroxylation of vitamin D3 in the liver (*34*, *35*), suggesting that the level of activated vitamin D and its downstream signaling may be increased. Considering the beneficial effects on health, typified by bone health, and the growth-promoting effects of vitamin D, its increase seems reasonable (*36*, *37*). Since vitamin D signaling has been suggested to contribute to the maintenance of the quiescence of muscle satellite cells (*38*), increased vitamin D signaling may explain the increased number of satellite cells and increased expression of its marker genes in the skeletal muscle of germ cell–removed males (Fig. 4, A and B). Consistent with this, the expression of *vdr*, a known vitamin D target gene, was significantly up-regulated in the skeletal muscles of germ cell–removed males (Fig. 4G) (*39*). In addition, vitamin D signaling may be increased in the skin of germ cell–removed males because vitamin D treatment has been reported to increase collagen production in dermal fibroblasts (*40*). Consistently, the expression of *cyp24a1*, a target of vitamin D, tended to increase in the skin of germ cell–depleted males (Fig. 4H). By contrast, in females, although the expression of *cyp2r1* increased in the liver, that of vitamin D target genes, *vdr* in the muscle and *cyp24a1* in the skin, were unchanged (fig. S7, A and B), suggesting that up-regulation of vitamin D signaling was marginal. Thus, although the expression of *pax7* in female muscle and *col1a1a* in female skin tended to increase, these changes could be caused by other signaling pathways in females (fig. S7, C and D). Together, these data suggest that increased vitamin D signaling may be involved in the improvement of the male life span caused by germ cell removal.

Last, the effects of vitamin D supplementation on somatic growth and maintenance were examined. To this end, we treated *N. furzeri* with alfacalcidol (alfa), an analog of vitamin D. We confirmed that alfa treatment increased the expression of vitamin D target genes (fig. S8). Similar to the results of a previous zebrafish study (*37*), alfa-treated *N. furzeri* males exhibited an increased body size (Fig. 4, I and J, and fig. S9, A and B). We also found that the thickness of the collagen fiber area and the expression of *col1a1a* in the skin were increased with alfa treatment (Fig. 4, K and L). Although vitamin D treatment has been suggested to have beneficial health effects (*36*), its effects on organismal aging in vertebrates remain elusive. We found that alfa treatment significantly improved the survival rate in both males and females (Fig. 4, M and N, and fig. S9, C and D), suggesting that activation of vitamin D signaling improves health at the organismal level. Thus, activation of vitamin D signaling could increase somatic growth and prevent aging in vertebrates.

## DISCUSSION

In general, reproduction is thought to be negatively correlated with life span. Germ cell removal in *C. elegans* provides not only consistent results with this idea but also a new insight that specific molecular signals connect the germ and soma (*9*, *10*). In this study, using a vertebrate model, it was further revealed that different signaling pathways interconnect germ cells, somatic growth, and aging in different ways depending on the sex. This might reflect the complexity of functional differences in vertebrate gonads between sexes.

Germ cell removal shortened the life span of female *N. furzeri*. A reduction in estrogen can explain this result (*22*). However, the mechanism by which germ cells regulate estrogen production remains unclear. The effects of germ cell removal on steroidogenesis vary depending on conditions and species. The defective steroidogenesis caused by germ cell removal observed in this study is similar to that observed in *dnd*-knockout Atlantic salmon (*41*). In contrast, estradiol production is not affected in *Dazl* knockout mice, in which oocytes are completely ablated in the adult ovaries (*42*). Given that undifferentiated germ cells are present at least until after birth, whereas germ cells in *dnd*-deficient animals are ablated during embryogenesis (*43*), the difference in the timing of germ cell ablation may explain the different effects on steroidogenesis. Although controversial, considering the association between oocyte loss and low estrogen levels during menopause (*44*), conserved mechanisms connecting germ cells to estrogen production may exist.

Increased IGF-1 signaling in germ cell–removed females may underlie increased body size and accelerated aging, although the mechanisms underlying the up-regulation of *igf1* expression are unclear. Since the levels of both estradiol and 11-ketotestosterone were very low in germ cell–removed females, they may have remained sexually immature. Considering that serum IGF-1 levels continue to increase until maturation in humans (*45*), the increased *igf1* might be associated with the possible absence of sexual maturation.

The increase in both the body size and life span of male germ cells may be caused by the increased signaling activity of vitamin D, an endocrine steroid hormone. In *C. elegans*, life-lengthening signals up-regulated by germ cell removal include dafachronic acid, a steroid hormone, and its receptor abnormal DAuer Formation (DAF)–12, a homolog of vertebrate vitamin D receptors (*10*), suggesting a possible evolutionary link between vertebrates and *C. elegans*. Although underlying mechanisms of increased vitamin D signaling by germ cell removal is a mystery at present, the life-lengthening effect of vitamin D observed in this study further supports the prospective role of vitamin D as a life-span–extending hormone (*36*). Given the moderate increase in vitamin D target gene expression in the tissues of male germ cell–removed mice, vitamin D signaling may be mildly increased. Although our results are apparently contradictory in that aberrant activation of vitamin D signaling rather shortened life span in α*-Klotho* knockout mice (*46*), this inconsistency would be due to the activation levels of vitamin D signaling, and mild activation seems to be beneficial to health. The actual mechanism by which vitamin D increases body size remains unknown (*37*). Further investigation of the effects of vitamin D signaling would lead to a better understanding of the mechanisms underlying improved somatic growth and slowed aging.

This study demonstrated the sex-dependent roles of germ cells in the regulation of life span and body size in *N. furzeri*. The sex difference may underlie the evolution of different reproductive strategies between sexes: Males are often selected to pursue “live fast, die young” reproductive strategy characterized by more rapid aging compared to females, although many exceptions to this pattern are observed (*47*). Further examinations of other species are necessary to better understand the conservation and diversity of the mechanisms.

## MATERIALS AND METHODS

### Ethical approval

All experimental animal care was performed in accordance with the institutional and national guidelines and regulations. The study protocol was approved by the Institutional Animal Care and Use Committee of Osaka University (RIMD permit #R02-04). The study was conducted in accordance with the ARRIVE (Animal Research: Reporting of In Vivo Experiments) guidelines.

### Fish strain, husbandry, and maintenance

The GRZ (GRZ-AD) strain of *N. furzeri* was donated by A. Antebi (Max Planck Institute for Biology of Aging). Fish were maintained at 26.5°C, 0.7 conductivity on a 12-hour light-dark cycle in the fish breeding system (Meito, Nagoya, Japan). For *dnd* MO, fish were raised at a density of one fish per 1.4-liter tank from the age of 2 weeks. For *dnd* MO-2, fish were raised at a density of three fish per 1.4-liter tank from 2 weeks to 1 month old and then raised at a density of 1 fish per 1.4-liter tank from 1 month old. The fish were fed freshly hatched brine shrimp twice a day from Monday to Saturday and once a day on Sunday. From the age of 2 weeks, the fish were fed bloodworms (Kyorin, Himeji, Japan) once a day.

### Generation of *vasa:EGFP* strain

A 2615-bp region of the *vasa* promoter and a 1964-bp region of the *vasa* 3′ untranslated region of *N. furzeri* were cloned next to EGFP. Plasmid DNA and Tol2 transposase mRNA were coinjected into one-cell stage fertilized eggs. Transgenic fish were outcrossed with wild-type fish to produce the founder line and maintained as homozygous transgenic fish.

### Generation of germ cell–removed *N. furzeri*

*dnd* MO (5′-TTAATAACATCTCACCTTCAGGAGG-3′) and *dnd* MO-2 (5′- TTATGGACTAATGCACCCAGCAGCA-3′), which are complementary to a splicing site for exon 1 and intron 1 and other region of exon 1 in *dnd* gene, respectively, were designed and obtained from Gene Tools (Philomath, OR, USA). Standard control morpholino (5′-CCTCTTACCTCAGTTACAATTTATA-3′) was used as a control. Approximately 1 nl of the solution containing MO (5 ng/μl) and 0.1% phenol red (Sigma-Aldrich, St. Louis, MO, USA, #P0290) was injected into the cell body of one-cell stage fertilized eggs. In *vasa*:EGFP, germ cell deficiency was confirmed by fluorescence stereomicroscopy at approximately 10 to 14 days postfertilization. In the wild type, germ cell deficiency was confirmed by observing morphological changes in the gonads when the fish were euthanized at the adult stage.

### Life-span measurement of germ cell–removed *N. furzeri*

Housing conditions were the same as those described above. The fish were fed freshly hatched brine shrimp twice a day from Monday to Saturday and once a day on Sunday. Fish were also fed bloodworms once a day from 2 weeks to 1 month of age and twice (*dnd* MO) or once (*dnd* MO-2) a day from 1 month of age. Fish mortality was documented daily from 1 month of age. Life-span analyses were performed using GraphPad Prism (GraphPad Software, San Diego, CA, USA) for all survival curves using the Kaplan-Meier estimator. To compare the survival curves between different experimental groups, we performed a log-rank test to examine whether the survival curves were significantly different. In males injected with *dnd* MO, the life span was not significantly changed, as evidenced by the log-rank test, likely because the mortality rate of germ cell–removed males caught up with that of control at a very old age (~4.5 months). Thus, to analyze the difference at old age (3 months), a comparison of survival rate at a fixed time point (at 100 days) was performed using the R package, “ComparisonSurv” (*48*).

### Genotyping of the sex-determining gene of *N. furzeri*

A 9-bp deletion in *gdf6Y* was examined by PCR using the primers listed in table S3. The PCR fragments were analyzed using a microchip electrophoresis system (MCE-202 MultiNA; Shimadzu, Kyoto, Japan) according to the manufacturer’s instructions using a DNA-500 reagent kit (Shimadzu).

### Measurement of steroid levels using liquid chromatography with tandem mass spectrometry

Frozen samples in 2.0-ml plastic tubes were homogenized in 700 μl of cold methanol with zirconia beads using a freeze crusher (TAITEC Corp., Koshigaya, Japan) at 41.6 Hz for 4 min. The homogenates were mixed with 800 μl of methanol and vortexed for 20 min at room temperature. The samples were centrifuged at 15,000 rpm (20,000*g*) for 15 min at 4°C, and the supernatants were collected. The insoluble pellets were further extracted with 800 μl of methanol and 800 μl of CHCl3. After centrifugation, the supernatants were collected and combined in a 15-ml tube. The samples were mixed with 800 μl of H_2_O and centrifuged at 4400 rpm (3000*g*) for 10 min at room temperature. The organic phase was collected and dried in a vacuum concentrator. The dried material was redissolved in 400 μl of CHCl3 and mixed with 5 ml of hexane. The samples were loaded onto ISOLUTE SI columns (Biotage, Uppsala, Sweden) and prewashed with methanol and hexane. After sample loading, the columns were washed with 2 ml of hexane. Samples were eluted with 3 ml of acetone. The eluates were dried, redissolved in 50% acetonitrile, and analyzed by liquid chromatography with tandem mass spectrometry.

Chromatographic separation was performed on an ACQUITY BEH C18 column (50 mm by 2.1 mm, 1.7-μm particles; Waters, Milford, MA, USA) in combination with a VanGuard precolumn (5 mm by 2.1 mm, 1.7-μm particles) using an Acquity UPLC H-Class System (Waters). The mobile phase consisted of solvent A, 0.1% formic acid in acetonitrile, and solvent B, 0.1% formic acid in H_2_O, and was delivered at a flow rate of 0.3 ml/min at 40°C. Linear gradients were as follows: 40% A at 0 to 0.5 min, 40 to 100% A at 0.5 to 2 min, 100% A at 2 to 3.5 min, and 40% A at 3.5 to 6 min. Mass spectrometric analysis was conducted using a Xevo TQD triple quadrupole mass spectrometer (Waters) coupled with an electrospray ionization source in the positive ion mode. The peak area of target steroids was analyzed using MassLynx 4.1 software (Waters). The analytical conditions were optimized using standard reagents obtained from Sigma-Aldrich and Wako (Osaka, Japan). The obtained signals were normalized to the wet weights of the corresponding samples.

### RNA-seq: Bulk RNA barcoding and sequencing

Tissue samples were homogenized in TRIzol reagent (#15596018; Invitrogen, Waltham, MA, USA), and total RNAs were extracted using the RNA Clean & Concentrator kit (#R1017; Zymo Research, Irvine, CA, USA). Tissues from five individuals were used for the preparation of five independent libraries. The bulk RNA barcoding and sequencing (*49*) was performed for library preparation with some modifications. Barcoded dT primer [5′-GCCGGTAATACGACTCACTATAGGGAGTTCTACAGTCCGACGATCNNNNNNN-NNNCCCCCCCCCTTTTTTTTTTTTTTTTTTTTTTTTV-3′; (10) N = unique molecular identifier (UMI), (9) C = cell barcode] was used for reverse transcription. A second-strand synthesis module (#E6111, New England Biolabs, Ipswich, MA, USA) was used for double-stranded cDNA synthesis. In-house MEDS-B Tn5 transposase (*50*, *51*) was used for tagmentation, and libraries were amplified by 10 cycles of PCR using Phusion High-Fidelity DNA Polymerase (#M0530; Thermo Fisher Scientific, Waltham, MA, USA) with the following primers: 5′-AATGATACGGCGACCACCGAGATCTACACindexGTTCAGAGTTCTACAGTCCGA-3′ and 5′-CAAGCAGAAGACGGCATACGAGATindex GTCTCGTGGGCTCGGAGATGT-3′. A 19-bp barcode read (Read1) and an 81-bp insert read (Read2) were obtained on an Illumina NovaSeq6000 (Illumina, San Diego, CA, USA).

### RNA-seq: Data processing

Fastq files of the liver and muscle samples were demultiplexed by deML (v1.1.3), and for liver (female) and muscle samples, 1M reads were randomly sampled from each fastq file obtained by using seqtk version: 1.3. Read1 (barcode read) was extracted using UMI-tools (ver.1.1.2) with the following command: “umi_tools extract -I read1.fastq --read2-in=read2.fastq --bc-pattern=NNNNNNNNNNCCCCCCCCC --read2-stdout.” Adaptor sequences and low-quality sequences were removed, read lengths below 20 bp were discarded using Trim Galore (ver.0.6.7), and the reads were mapped to the *N. furzeri* GRZ reference genome [GRZ Assembly (May 2015); https://nfingb.leibniz-fli.de/] using HISAT2 (ver.2.2.1). Read counts for each gene were obtained by featureCounts (ver.2.0.1), and UMI duplication was removed by UMI-tools with the following command: “umi_tools count --method=unique --per-gene --per-cell --gene-tag=XT.” A pairwise differential expression analysis was performed using the quasi-likelihood method of Degust ver.4.1 (*52*) (quasi-likelihood methods in edgeR). The GO enrichment analysis was performed using ShinyGO 0.77 (*53*). Gene IDs were converted to Ensembl gene IDs for zebrafish before GO enrichment analysis.

### Reverse transcription polymerase chain reaction

For qPCR analysis, cDNA was synthesized using the ReverTra Ace qPCR RT master mix with genomic DNA remover (#FSQ-301; Toyobo, Osaka, Japan). qPCR analysis was conducted on a Stratagene Mx3000P qPCR system using THUNDERBIRD SYBR qPCR Mix (#QPS-201; Toyobo) using the following conditions: 95°C (1 min), 45 cycles at 95°C (15 s) and 60°C (35 s). A standard curve was used to determine the relative mRNA abundance. The *tbp* or *actb* (fig. S1, B and C) genes were used as a normalization control.

For the RT-PCR analysis of gonadal genes shown in Fig. 2B, cDNA was synthesized using the Transcriptor High Fidelity cDNA Synthesis Kit (#5091284001; Roche, Basel, Switzerland). RT-PCR was performed using Ex Taq (#RR001; Takara, Kusatsu, Japan) at the following conditions: 94°C (5 min), 25 (for *actb*) or 32 (for other genes) cycles at 94°C (30 s), 55°C (30 s), and 72°C (10 s), ending with a 3-min extension phase at 72°C. The PCR products were run on a 2% agarose gel. The primers used are listed in table S3.

### Histology

Tissue samples were fixed with 4% paraformaldehyde in phosphate-buffered saline (PBS) at 4°C overnight. They were placed in 10 and 20% sucrose/PBS until the tissues sank and 30% sucrose/PBS overnight at 4°C. Tissues were embedded in a Tissue-Tek optimal cutting temperature (OCT) freezing medium (Sakura Finetek, Tokyo, Japan). Slices (10-μm thick) were prepared using a Thermo Fisher Scientific HM525NX cryostat and stored at −80°C until used.

### Oil Red O staining

The sections were immersed in propylene glycol for 5 min. Then, they were incubated in heated (60°C) Oil Red O solution (#ORG500; Scy Tek, Logan, UT, USA) for 10 min. They were lastly differentiated in 85% propylene glycol for 1 min and counterstained with hematoxylin. Images were acquired using a BZ9000 (Keyence, Osaka, Japan) (for Fig. 3D) and an IX73 (Olympus, Tokyo, Japan) (fig. S5A) microscope in the bright field using a 20× objective lens. Images of two or three randomly selected regions of liver sections were obtained, and the average quantified values were computed for each section. Quantification was performed using the ImageJ software. Oil Red O–stained area was extracted with “Color Transformer.” Subsequently, the signal was binarized with an automated thresholding method, and the area of the signal was determined.

### SA-βgal staining

Staining was performed as previously described (*29*). Briefly, sections were stained in X-Gal staining solution {7.37 mM citric acid, 25.26 mM Na phosphate, 5 mM K_4_[Fe(CN)_6_] 3H_2_O, 5 mM K_3_[Fe(CN)_6_], 150 mM sodium chloride, 2 mM magnesium chloride, and X-gal [1 mg/ml] in distilled water} for 1 to 2 hours at 37°C and counterstained with Nuclear Fast Red (#NFS500; Scy Tek). Images were acquired using an IX73 (Olympus) microscope in the bright field using a 20× objective lens. Images of three randomly selected regions of liver sections were obtained, and the average quantified values were used for each section. Quantification was performed using the ImageJ software. The SA-βgal–stained area was extracted with a Color Transformer. Subsequently, the signal was binarized with an automated thresholding method, and the area of the signal was determined.

### Masson’s trichrome staining

Sections were fixed with Bouin Solution (#023-17361; Wako) for 1 hour at 55°C. Staining was performed using a trichrome staining (Masson) kit (#HT15-1 KT; Sigma-Aldrich). Images were acquired using an IX73 (Olympus) microscope in the bright field. A 10× objective lens (Fig. 4C) and a 4× objective lens were used (Fig. 4K and fig. S6). Quantification was performed using the ImageJ software. The area of collagen fiber was extracted with Color Transformer [left or right side of the trunk (Fig. 4C) and both sides of the trunk (Fig. 4K and fig. S6)]. Subsequently, the signal was binarized with the automated thresholding method, and the area of the signal was measured. Last, the thickness was calculated by dividing the measured area by the length of the outline of the trunk.

### Immunohistochemistry analysis

The sections were autoclaved in Target Retrieval Solution, Citrate (pH 6) (Dako, Glostrup, Denmark) for 15 min at 105°C for antigen retrieval. Sections were blocked with 10% fetal bovine serum/0.2% Triton X-100/1% dimethyl sulfoxide in PBS for 1 hour at room temperature. The primary antibody used was anti-Pax3/7 (DP312) (1:200) (*54*). The secondary antibody used was an anti-mouse immunoglobulin G antibody (#A32728, Invitrogen). Sections were mounted with VECTASHIELD Vibrance Antifade Mounting Medium with 4a,6-diamidino-2-phenylindole (#H-1800, Vector Laboratories, Newark, CA, USA). Confocal images were acquired using an FV-3000 confocal laser scanning microscope (Olympus) with a 10× objective lens. Images of the 1- to 1.5-mm^2^ region next to the neural tube were acquired. Images were analyzed with the FV31S-SW software. The number of Pax3/7+ nuclei was counted manually.

### Measurement of blood LDL and VLDL levels

Blood (5 to 20 μl) was collected in 20 mM EDTA (pH 8.0)/PBS. Blood LDL and VLDL levels were measured using an EnzyChrom AF HDL and LDL/VLDL assay kit (#E2HL-100; BioAssay Systems, Hayward, CA, USA) and normalized to the total protein level measured using the Bradford protein assay kit (#T9310A; Takara).

### Micro-CT analysis

Fish were fixed with 70% ethanol. The micro-CT analysis was performed with a ScanXmate-S090R (Comscan, Yokohama, Japan). The following settings were used: voltage, 45 kV; current, 180 μA; and magnification, ×4.9439, using a brass filter. Image data were reconstructed using the TRI/3D-BON software (Ratoc System Engineering, Kyoto, Japan). Bone volume and bone mineral density of nine vertebrae (including vertebral body, neural spine, and hemal spine) from the caudal section were measured.

### Alfa treatment

For life-span analysis and body size measurements in Fig. 4 (J, M, and N) and fig. S9D, approximately 60 embryos were hatched in a 3.6-liter tank. They were raised at a density of three fish per 1.4-liter tank from 2 weeks to 1 month old and then raised at a density of 1 fish per 1.4-liter tank from 1 month old. From one and a half months of age, 16 to 18 fish, including both males and females, were maintained in a 40-liter tank. The fish were incubated in the aquarium water with dimethyl sulfoxide or 5 nM alfa and fed Artemia twice daily. Twenty liters of the fish water was changed every alternate day. This treatment was performed in three independent tanks for both control and alfa. After 11 weeks of treatment, the fish were maintained in a normal breeding system with running water.

For life-span analysis and body size measurements in fig. S9 (B and C), approximately 60 embryos were hatched in a 3.6-liter tank and raised at a density of approximately 20 fish per 3.6-liter tank from 2 weeks to 1 month old. From 1 month of age, approximately 15 fish, including both males and females, were maintained in a 9-liter tank. The fish were incubated in the aquarium water with dimethyl sulfoxide or 10 nM alfa and fed Artemia twice daily. The fish water was changed every alternate day. After 1 month of treatment, the fish were maintained in a normal breeding system with running water. For Masson’s trichrome staining (Fig. 4K), the fish were hatched and raised in the same manner until 3 weeks of age. Fish were maintained at a density of approximately 10 fish per 3.6-liter tank until 1.5 months old. Fish were treated with alfa in the same manner, but each fish was maintained separately in a 9-liter tank with partitions. After 1 month of treatment, the fish were maintained in a normal breeding system with running water at a density of 1 fish per 1.4-liter tank. For RT-qPCR analysis, the fish were hatched in the same manner. They were raised at a density of three fish per 1.4-liter tank from 2 weeks to 1 month old and then raised at a density of 1 fish per 1.4-liter tank from 1 month old. At one and a half months of age, the fish were incubated in the aquarium water at a density of 4 to 5 fish per 3.6-liter tank with dimethyl sulfoxide or alfa (10 or 30 nM) and fed Artemia once daily for 3 days. The fish water was unchanged for Fig. 4L and fig. S8A, and 2 liters of the water was changed every day for fig. S8B.
