# Supplementary material for: Sex-dependent regulation of vertebrate somatic growth and aging by germ cells
Source: Sci Adv. 2024 Jun 12;10(24):eadi1621. doi: 10.1126/sciadv.adi1621 (PMC11168456; doi:10.1126/sciadv.adi1621)
Supplement: Supplementary file 1 — Figs. S1 to S12 Legends for data files S1 to S4 [file sciadv.adi1621_sm.pdf]

Science Advances
AAAS

Supplementary Materials for

Sex-dependent regulation of vertebrate somatic growth and aging by germ cells

Kota Abe *et al.*

Corresponding author: Tohru Ishitani, ishitani@biken.osaka-u.ac.jp

Sci. Adv. **10**, eadi1621 (2024)
DOI: 10.1126/sciadv.adi1621

The PDF file includes:

Figs. S1 to S12
Legends for data files S1 to S4

Other Supplementary Material for this manuscript includes the following:

Data files S1 to S4

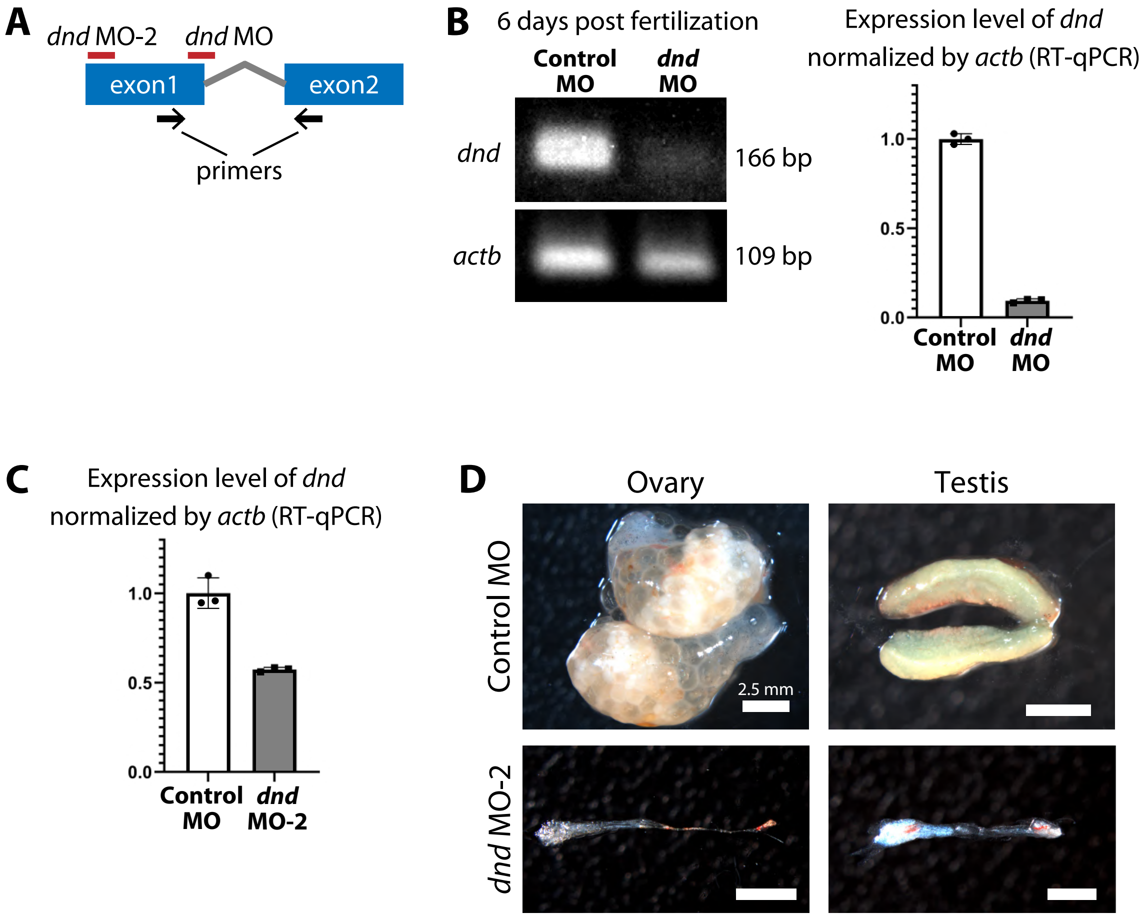

Fig. S1. Validation of morpholino to *dnd*. (**A**) Schematic illustration of the genomic structure of *dnd* gene. The *dnd* MO annealing sites are indicated by the red line. The primers used for validation are indicated by arrows. (**B** and **C**) RT-PCR and qPCR analyses of the *dnd* transcript using primers indicated in (A). RNAs were extracted from embryos injected with control MO, *dnd* MO (**B**), or *dnd* MO-2 (**C**) at 6 days post fertilization. A 166-bp band that corresponds to the normal transcript was produced in control but was reduced by the injection of *dnd* MOs. *actb* is used as the internal control. Bars and error bars represent mean ± SD of technical triplicate. (**D**) Gonads of *N. furzeri* injected with control MO or *dnd* MO-2.

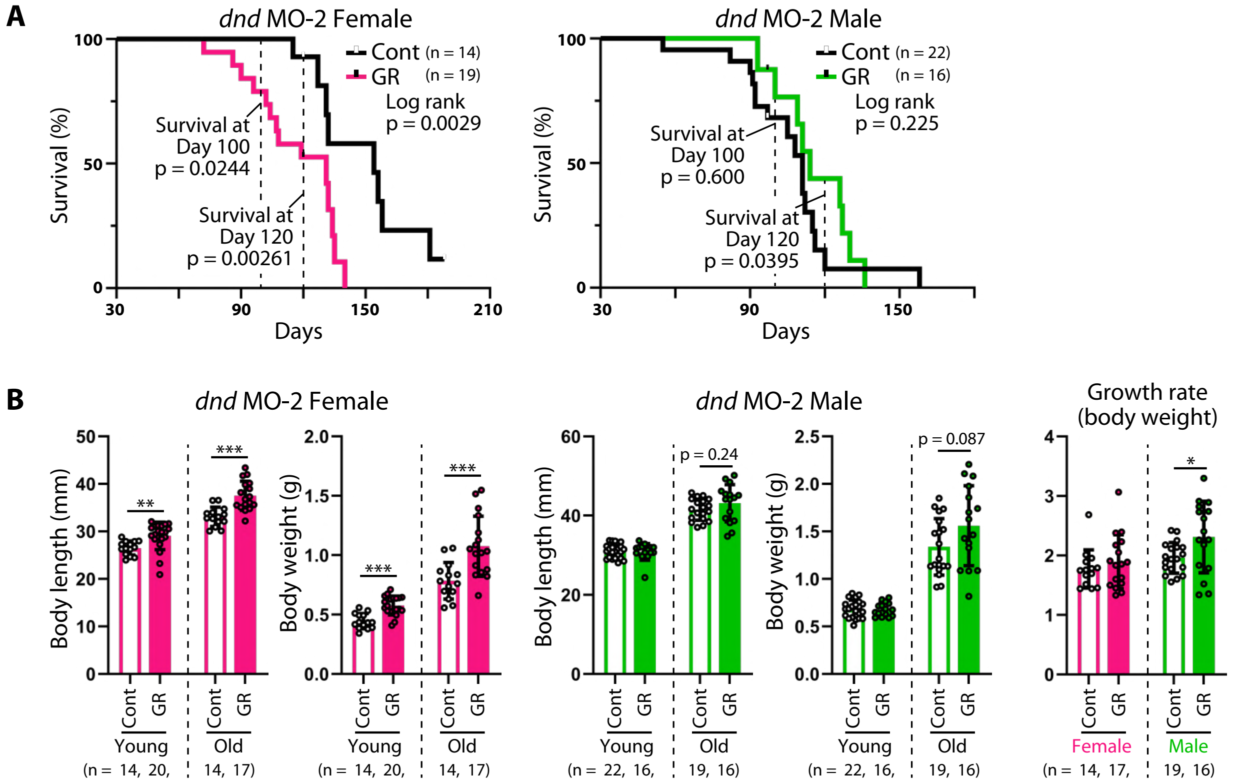

Fig. S2. *dnd* MO-2 showed similar effects on lifespan and body size to those by *dnd* MO. (**A**) Lifespan of control and germ-cell-removed *N. furzeri* injected with MO-2. (Left) Survival curves of control (Cont, black) versus germ-cell-removed female (GR, magenta). (Right) Survival curves of control (Cont, black) versus germ-cell-removed male (GR, green). (**B**) Quantification of body length and body weight of the control (Cont) and germ-cell-removed (GR) *N. furzeri* injected with *dnd* MO-2. The same individuals (excluding dead animals in old age) are used. The growth rate was calculated by dividing the body weight at the old age by that of the same individual at a young age. *P*-values are from Welch's t-test (**P* < 0.05; ***P* < 0.01; ****P* < 0.001). Bars and error bars represent mean ± SD.

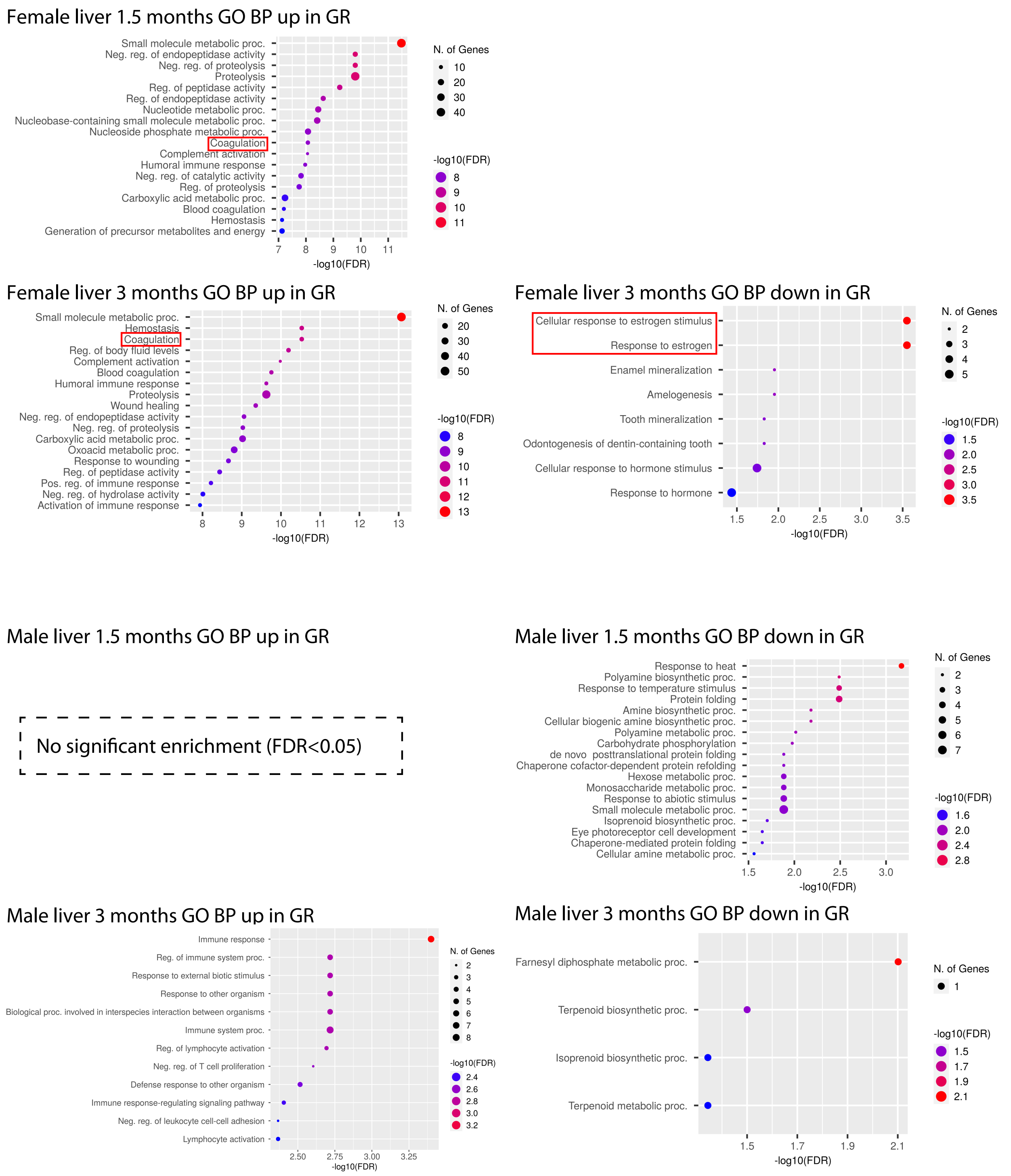

Fig. S3. Germ-cell-removal downregulates estrogen signaling and upregulates the expression of coagulation-related genes in females but not in males. Gene ontology (GO)-annotation analysis of the upregulated genes in the liver. BP, biological process. Cont, control. GR, germ-cell-removed. Red box indicates "Coagulation", "Cellular response to estrogen stimulus" and "Response to estrogen".

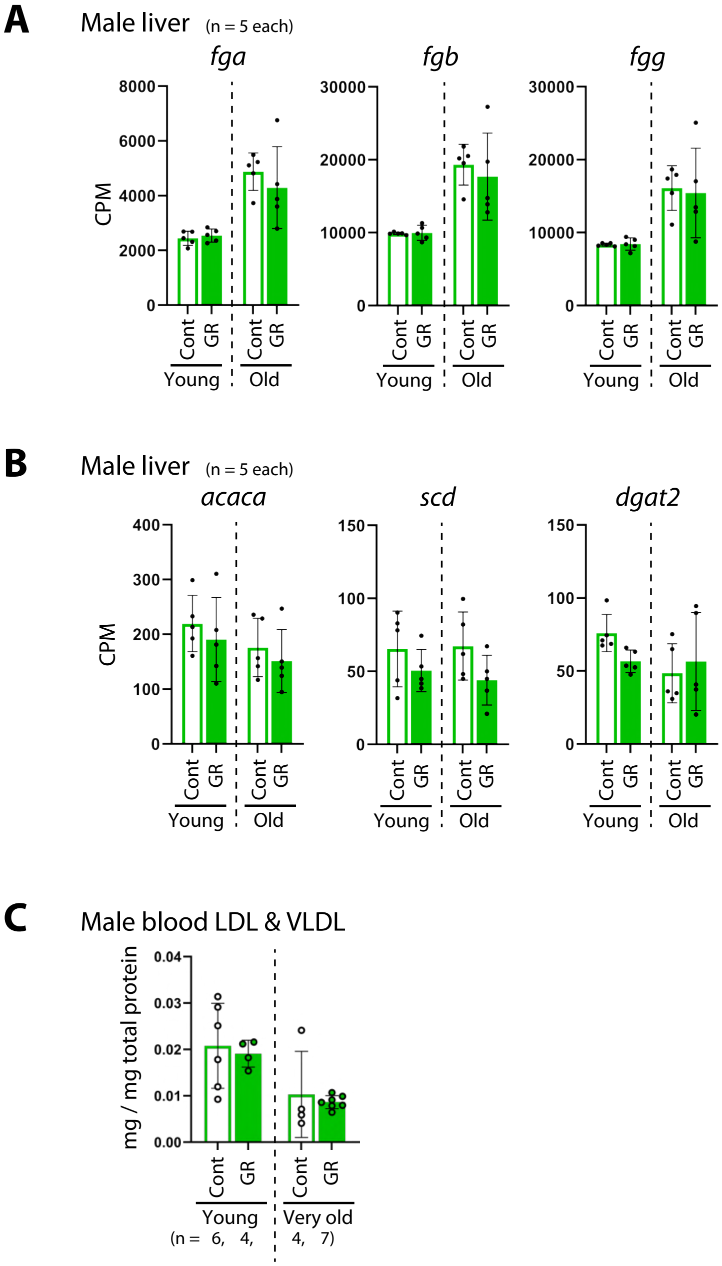

Fig. S4. Germ-cell-removal does not affect the expression of genes involved in coagulation, fatty acid biosynthesis, triglyceride biosynthesis in the male liver, and LDL and VLDL levels in the male blood. (**A**) Expression of fibrinogens, *fga*, *fgb*, and *fgg,* in the male liver by RNA sequencing (RNA-seq) analysis. (**B**) Expression of key genes involved in fatty acid biosynthesis (*acaca*) and triglyceride biosynthesis (*scd* and *dgat2*) in the male liver by RNA-seq analysis. CPM, count per million. (**C**) The sum of blood low-density lipoprotein (LDL) and very-low-density lipoprotein (VLDL) levels in males. Bars and error bars represent mean ± SD. Cont, control. GR, germ-cell-removed.

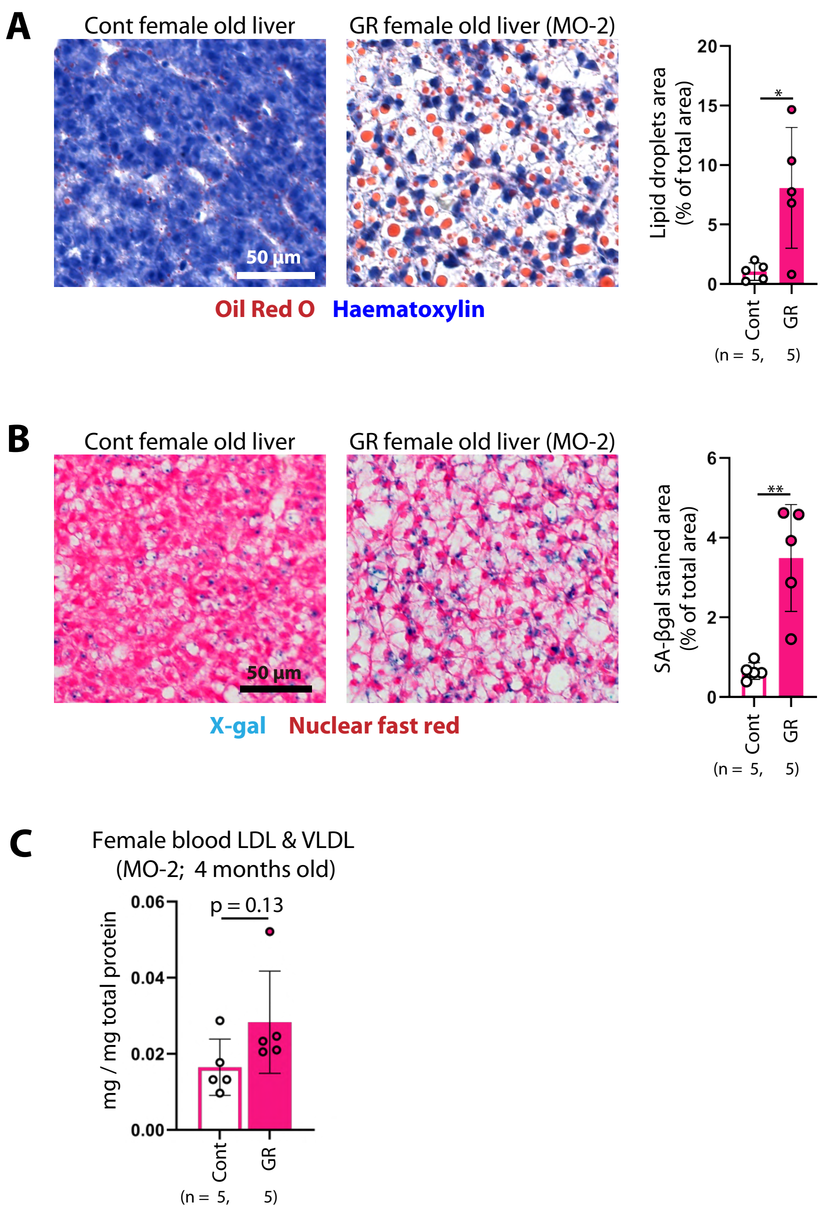

Fig. S5. Phenotypes in the female liver and blood injected with *dnd* MO-2 are similar to those injected with *dnd* MO. (**A**) Liver sections of old (3 months old) females stained with Oil Red O and counterstained with haematoxylin. (Right) Quantification of lipid accumulation in the liver. (**B**) Liver sections of old females (three months old) stained with senescence-associated beta-galactosidase (SA-βgal) and counterstained with nuclear fast red. (Right) Quantification of SA-βgal-stained area in the liver. (**C**) Sum of blood low-density lipoprotein (LDL) and very-low-density lipoprotein (VLDL) level in females at 4 months old. *P* values are from Welch's t-test (**P* < 0.05; ***P* < 0.01) in (A, B, and C). Cont, control. GR, germ-cell-removed.

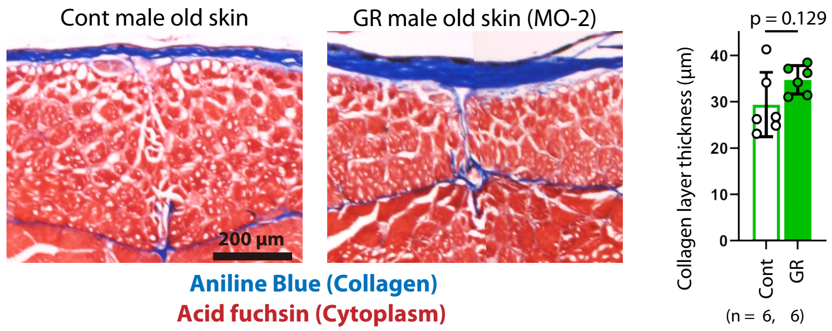

Fig. S6. Skin phenotype of males injected with *dnd* MO-2 is similar to that injected with *dnd* MO. Skin sections of old (three months) males injected with *dnd* MO-2 stained with Masson's trichrome stain. (Right) Quantification of the thickness of skin collagen fiber. *P* value is from Welch's t-test. Cont, control. GR, germ-cell-removed.

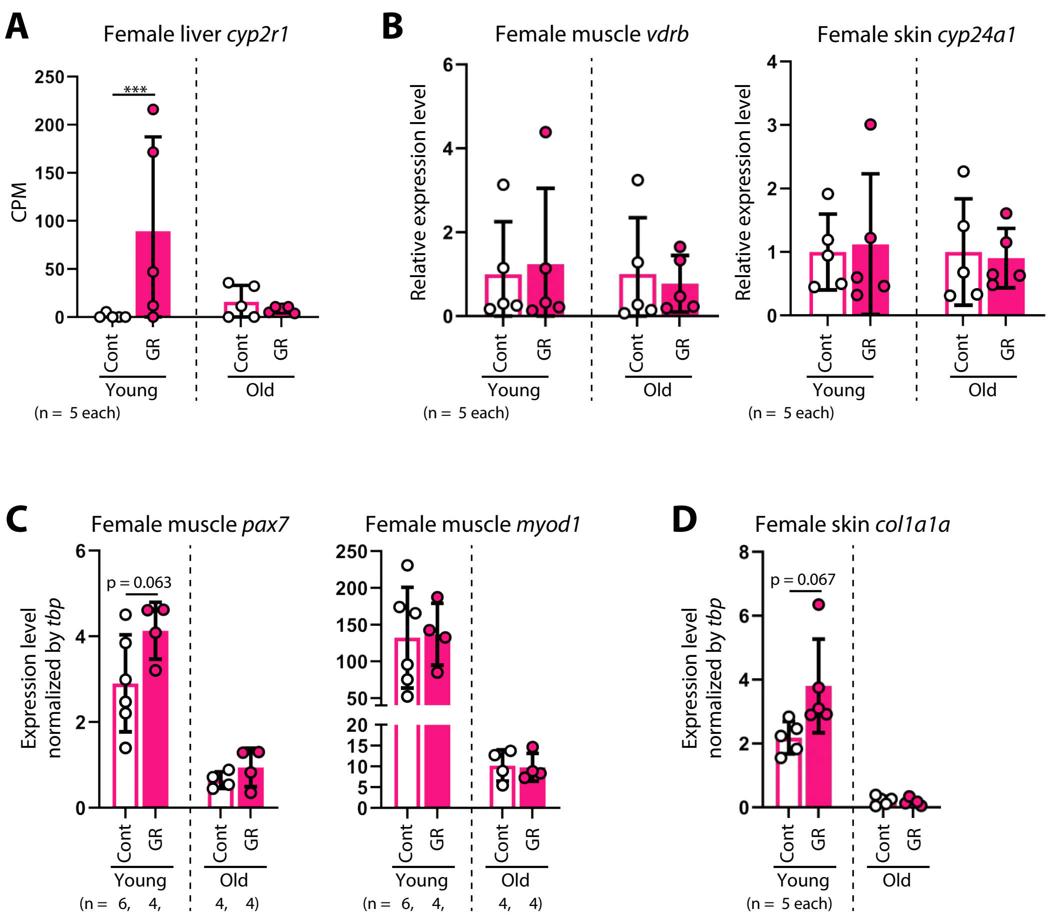

Fig. S7. Germ-cell-removal does not affect vitamin D signaling in the female muscle and skin. (**A**) Expression of vitamin D-activating enzyme, *cyp2r1*, in the female liver at young (1.5 months) and old (three months) age by RNA-seq analysis. CPM, count per million. FDRs are from quasi-likelihood methods in edgeR (***FDR < 0.001). (**B**) Expression of vitamin D signaling target gene, *vdrb* in the female skeletal muscle and *cyp24a1* in the female skin at young (1.5 months) and old (three months) ages by RT-qPCR analysis. (**C**) Expression of muscle satellite cell marker genes (*pax7* and *myod*) in the female skeletal muscle by RT-qPCR analysis. (**D**) Expression of *col1a1a* in the female skin at young (1.5 months) and old (three months) ages by RT-qPCR analysis. Bars and error bars represent mean ± SD. *P* values are from Welch's t-test in (C and D). Cont, control. GR, germ-cell-removed.

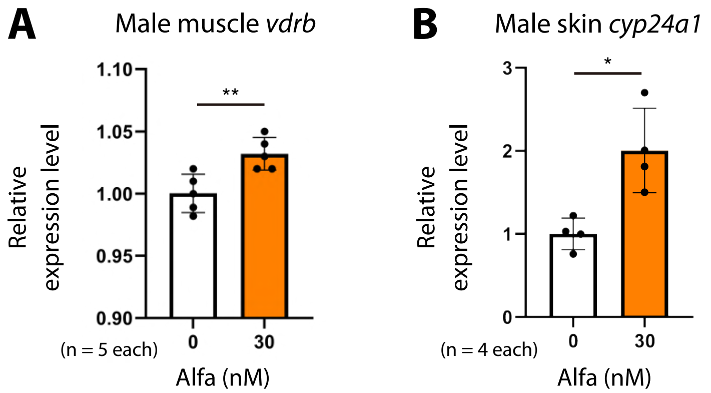

Fig. S8. Treatment with alfacalcidol (alfa) increased the expression of vitamin D target genes. (**A**) Expression of *vdrb* in the muscle of alfa-treated male at a young age by RT-qPCR analysis. (**B**) Expression of *cyp24a1* in the skin of alfa-treated male at a young age by RT-qPCR analysis. *P* values are from Welch's t-test (*$P < 0.05$; **$P < 0.01$).

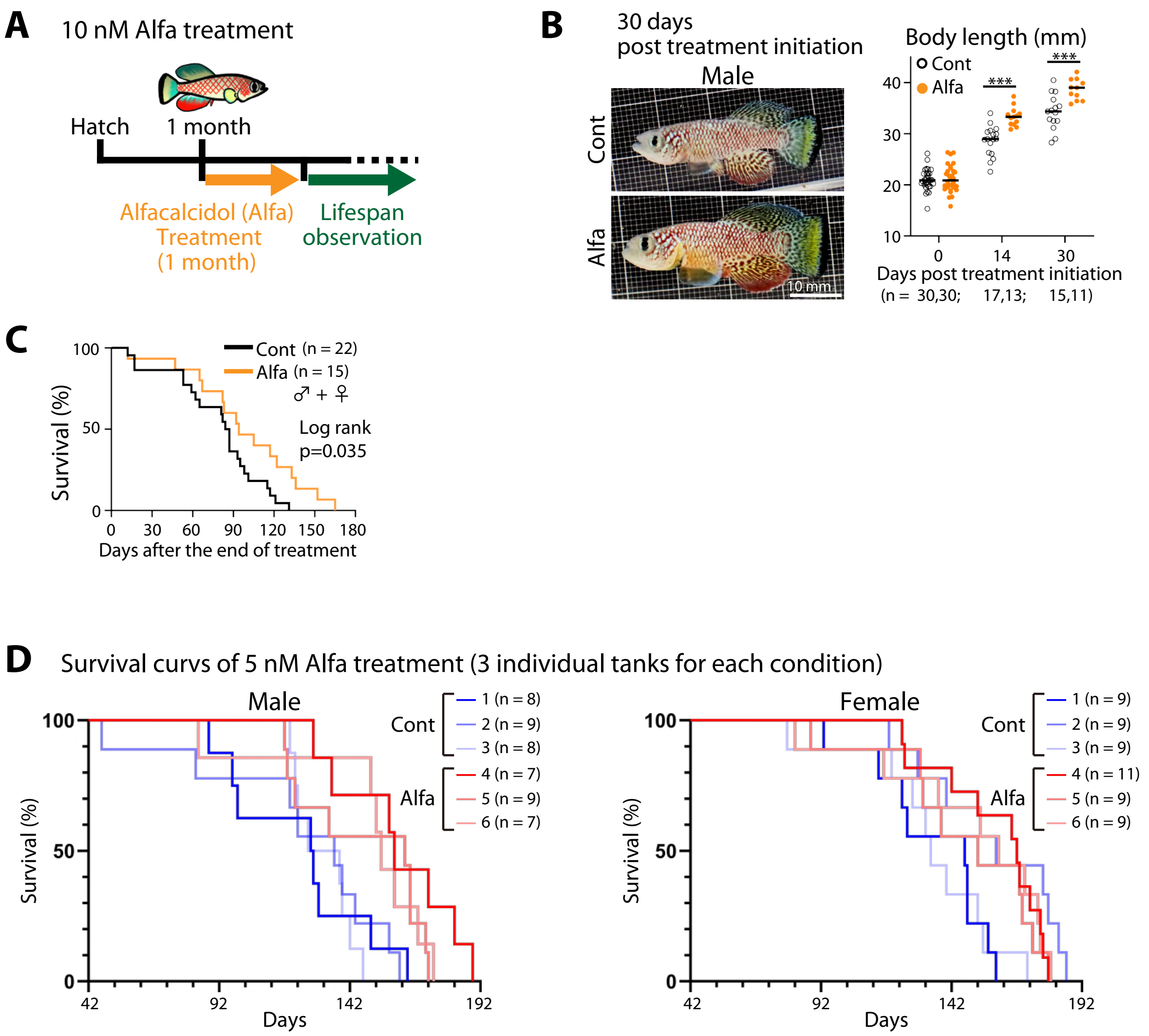

Log rank p-values (Male)

	1	2	3
4	0.0144	0.0286	0.0073
5	0.0412	0.0496	0.0848
6	0.0515	0.0637	0.0026

Log rank p-values (Female)

	1	2	3
4	0.0051	0.3087	0.0187
5	0.0838	0.2142	0.1907
6	0.0325	0.2855	0.0641

Fig. S9. Treatment with alfacalcidol (alfa) reproducibly enhances somatic growth and extends lifespan. (**A**) Schematic illustration of treatment with alfa (10 nM). (**B**) Representative images of alfa-treated males 30 days after the start of the treatment. (Right) Quantification of the body length of alfa-treated males from 0 to 30 days after the start of the treatment. *P* values are from Welch's t-test (****P* < 0.001). (**C**) The lifespan of control and alfa-treated *N. furzeri*. Results for males and females are mixed. Survival curves of control (black) versus alfa-treated animals (Orange). (**D**) The lifespan of control and alfa (5 nM)-treated *N. furzeri* in three individual tanks (data sets are the same as Fig. 4M and 4N). Survival curves of controls (Blue lines; 1–3) versus alfa-treated animals (Red lines; 4–6). *P*-values from the log-rank test are listed in the table below the survival curves.

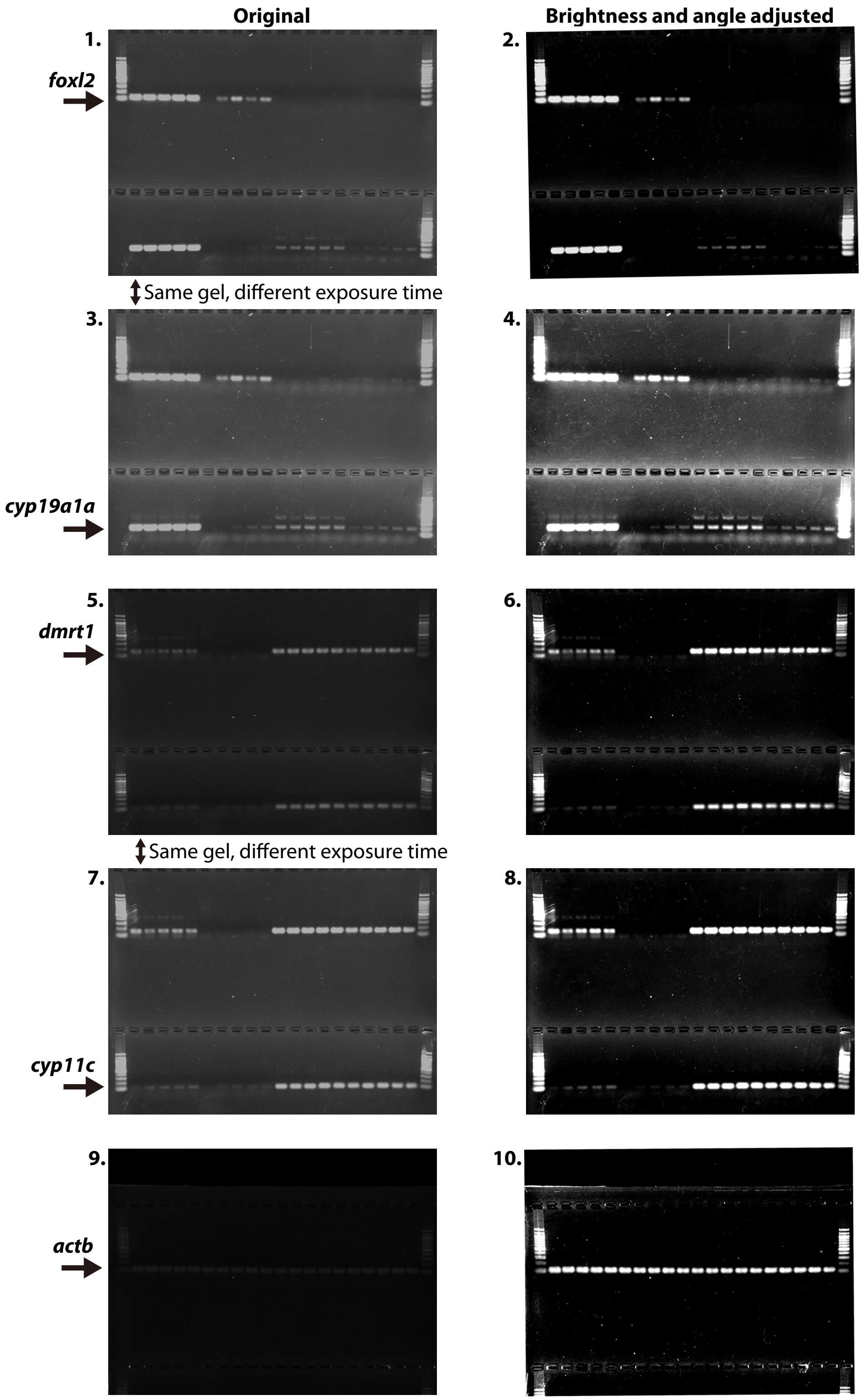

Fig. S10. Whole gel images of Fig. 2B. 1, 3, 5, 7, and 9 are original gel images for *foxl2*, *cyp19a1a*, *dmrt1*, *cyp11c*, and *actb*, respectively. 2, 4, 6, 8,10 are brightness and angle-adjusted images.

Supplementary Excel File 1. This file contains detailed information of GO analysis (statistics and gene names) in Fig. 3A and fig. S3.

Supplementary Excel File 2. This file contains lists of differentially expressed genes (FDR<0.05) between control and germ-cell-removed animals in the liver and old muscle from RNA-seq analysis.

Supplementary Excel File 3. This file contains lists of oligonucleotides for each of the experiments performed in this work.

Supplementary Excel File 4. This file contains lists of data values used in graphs.
